# LncRNA LINC00525 activates HIF-1α through miR-338-3p / UBE2Q1 / β-catenin axis to regulate the Warburg effect in colorectal cancer

**DOI:** 10.1080/21655979.2021.2018538

**Published:** 2022-02-13

**Authors:** Fanqi Meng, Xiaofan Luo, Chenyao Li, Guangyi Wang

**Affiliations:** a Department of Colorecal & Anal Surgery, The First Hospital of JiLin University Changchun, China; b Department of Hepatobiliary and Pancreatic Surgery, The First Hospital of JiLin University

**Keywords:** Colorectal cancer, HIF-1α, LINC00525, ubiquitination, Warburg effect

## Abstract

Warburg effect is considered to be related to the malignancy of tumor cells under hypoxic conditions, but the underlying mechanism remains unknown. In this article, it has been reported that lncRNA LINC00525 is a hypoxia-responsive lncRNA and is essential for hypoxia-enhanced glycolysis. It was found that LINC00525 was up-regulated, and promoted cell proliferation in colorectal cancer in vitro and in vivo. In colorectal cancer cells, hypoxia increasedLINC00525 expression, whereas knocking down LINC00525 reduced hypoxia-enhanced glycolysis. For specific molecular mechanisms, it was found that LINC00525 promoted UBE2Q1 expression by binding miR-338-3p, and UBE2Q1-stabilized β-catenin enhances hypoxia-enhanced glycolysis by activating HIF-1α. In conclusion, these findings showed that LINC00525 was essential for hypoxia-enhanced glycolysis; its mechanism was related to activating HIF-1α through miR-338-3p/UBE2Q1/β-catenin axis in colorectal cancer cells.

## 1 Introduction

Cancer cells can escape the normal apoptosis pathway by exhibiting a unique metabolic phenotype that enhances the proliferation and migration ability. This unique metabolic phenotype refers to the Warburg effect, and it plays a significant role in the pathogenesis of tumors [1]. The Warburg effect occurs when cancer cells weaken aerobic respiration through a series of molecular mechanisms under aerobic conditions, carry out efficient glycolysis, and then obtain a large amount of adenosine triphosphate, and create a microenvironment suitable for tumor cells to survive, proliferation and movement [2,3]. In addition, the Warburg effect inhibits the monitoring and lethality of T lymphocytes through local hypoxia, creating tumor immune escape [4]. In conclusion, the Warburg effect is a critical factor in tumor cell malignancy, but its exact molecular mechanism is unclear, and adaptive responses to the unique tumor hypoxic microenvironment may represent a well-known mechanism [5].

Long non-coding RNA (lncRNA) is a non-coding RNA with a length of more than 200nt, and was regarded as a ‘transcription byproduct’ or ‘junk gene’ in the long past years because it did not encode a protein [6]. With the advancement of gene sequencing technology in recent years, an increasing number of lncRNAs have been identified and identified as being related to the occurrence and progression of malignant tumors [6,7]. LncRNA LINC0052 is a newly discovered lncRNA that has been found to function as oncogenes in a variety of malignant tumors, including glioma [8], chordoma [9], non-small cell lung cancer [10], and colorectal cancer [11]. Importantly, previous studies reported that LINC00525 is highly expressed in colorectal cancer tissues, and is related to the poor prognosis of colorectal cancer patients [12], and promotes the proliferation of colorectal cancer cells, and inhibits apoptosis [11,13].

miR-338-3p has been found to function as a tumor suppressor gene in colorectal cancer [14,15], and can be used as a downstream target gene for a variety of lncRNAs to function as oncogenes to regulate biological characteristics of colorectal cancer cells [16,17], including LINC00525^10^. Moreover, miR-338-3p has also been found to be involved in the regulation of the Warburg effect of tumor cells [18], and as a downstream gene of lncRNA to promote glycolysis in tumor cells [19]. Ubiquitin-conjugating enzyme E2Q family member 1 (UBE2Q1), a target gene of miR-338-3p [20], has been found to stabilize β-catenin in hepatocellular carcinoma [21]. Due to β-catenin stabilized HIF-1α by interacting with HIF-1α under hypoxic condition [22] and HIF-1α was a key mediator of hypoxic response, UBE2Q1 might stabilize HIF-1α by stabilizing β-catenin. In this study, it was hypothesized that LINC00525 regulated the expression of UBE2Q1 is activated by miR-338-3p via the bridging effect, whereas UBE2Q1 activated HIF-1 via stabilizing-catenin, ultimately promoting hypoxia-enhanced glycolysis in colorectal cancer. The aim of the present study was to discover a new mechanism for LINC00525 to regulate hypoxic glycolysis in colorectal cancer cells. The goal of this study was to demonstrate that LINC00525 activated HIF-1α through miR-338-3p/UBE2Q1/β-catenin axis to regulate the Warburg effect in colorectal cancer.

## 2 Materials and methods

### 2.1 Cell culture and cell

Human normal colonic epithelial cells (NCM-460) and human colorectal cancer cells (SW480, HCT116, HT29, SW620, SW1116, and LoVo) were all cultured in McCoy’s 5A medium (12,330,031, Gbico) supplemented with 10% fetal bovine serum (16,140,071, Gbico) in 37°C with 5% CO2.

## 2.2 qPCR analysis

Cells were collected to extract total RNA using an RNA extraction Kit (RC101-01, Vazyme, China), and cDNA was produced as directed using a PrimeScript RT reagent (RR047A, Taraka, Japan) [23,24]. Then, 20 μL of the qPCR system was prepared and analyzed as described in the instructions of GoTaq qPCR Master Mix (A6001, Promega, USA) in a real-time quantitative PCR instrument(Accurate96-x4, Dlab). The relative expression of the gene was calculated by the 2^−ΔΔCt^ method. β-actin was loaded as a control for lncRNA and mRNA, and U6 was loaded as a control for miRNA. Primers for qPCR are shown in Table 1.

**Table 1.** Sequences of primers of qPCR, si-RNA and miRNA

Primers used in RT-qPCR analysis (5ʹ-3ʹ)	
LINC00525	F: CACACCCCAAGCAACAATCG
	R: GAGTGTTGGTGCAGTCCTCA
miR-338-3p	F: ACACTCCAGCTGGGTCCAGCATCAGTGATT
	R: TGGTGTCGTGGAGTCG
U6	F: CTCGCTTCGGCAGCACA
	R: AACGCTTCACGAATTTGCGT
β-actin	F: GGCTGTATTCCCCTCCATCG
	R: CCAGTTGGTAACAATGCCATGT
Si-RNA sequence (5ʹ-3ʹ)	
si-NC	GCAAAUGAAUACAACAAAAUA
si-525-1	AUUAAGGUUCUCAAUACAGUA
si-525-2	UAUAUAUUUUGUUGUAUUCAU
si-525-3	AUAUGCAUGAUUUCUUAGGUG
si-β-catenin	CAUUAGUUCAUUUCAUUCAUU
miRNA regulate sequence (5ʹ-3ʹ)	
miR-338-3p-nc	TAAGCGAATTCCGTGGTCTAAC
miR-338-3p-mimic	TCCAGCATCAGTGATTTTGTTG
miR-338-3p-inhibitor	AGGTCGTAGTCACTAAAACAAC

### 2.3 Cell transfection and adenovirus infection

To knock down the expression of LINC00525 and β-catenin, we transfected small interfering RNA into colorectal cancer cells as previously described [25,26]. In brief, 50 nmol/l of special small interfering RNA for LINC00525 (si-525) or β-catenin (si-β-catenin) were directly transfected into 2.5 × 10^6^ SW620, SW1116 and LoVo cells using Lipofectamine 2000 according to the manufacturer’s protocols. After 24 hours of transfection, experiments were performed. Similarly, 50 nmol/l of miR-338-NC (NC), miR-338-mimic (mimic), and miR-338-inhibitor (inhibitor) were directly transfected into 2.5 × 10^6^ SW620, SW1116 and LoVo cells using Lipofectamine 2000 according to the manufacturer’s protocols. To test the target binding between miR-338-3p and LINC00525/UBE2Q1, we first cloned wild-type (WT) or mutated (MUT) versions of the 3ʹ-UTR of LINC00525 or UBE2Q1 into pisCHECK2 (Addgene, 97,157) to build a recombinant plasmid. Next, LoVo cells expressing NC, mimic, or inhibitor was transfected with recombinant plasmid. To overexpress UBE2Q1 expression, 30 μl adenovirus for overexpressing UBE2Q1 (AAV-UBE2Q1) (Vector Biolabs, AAV-MI0012528) were directly added into the cell culture medium, and empty adenovirus (AAV-NC) was used as control.

### 2.4 FISH staining analysis

1 x 10^5^ cells were seeded into Lab-Tek cell culture well (155,411, Thermo Scientific). The cells were fixed with cold acetone 24 hours later and then incubated with a fluorescent probe for binding to the human version of the LINC00525 gene, which was synthesized by Genomeditech Co., Ltd. For cells, it should be counterstained the nucleus with 5 μg/mL DAPI for 5 minutes at room temperature. At last, all samples were analyzed by confocal microscopy (TCS SP5, Leica).

### 2.5 Cell proliferation assay

A total of 1 × 10^4^ cells were seeded into 96 cell culture plates and cultured for 72 hours at 37°C with 5% CO2. The cell viability was measured with CCK8 reagent (C0038, Beyotime) at 0, 24, 48, and 72 hours respectively.

For cell clone test: 200 cells with 2 mL medium were seeded into a 6-well cell culture plate and culture for 2 weeks at 37°C with 5% CO2. At last, we removed the cell culture medium and washed them twice with PBS buffer. Added formaldehyde to fix at room temperature for 30 minutes, and then add 0.1% crystal violet for staining at room temperature for 20 minutes and wash 3 with PBS. Finally, the cell clone number was counted.

In vivo cell proliferation experiment [27]: we injected 5 × 10^6^/0.2 ml cells into the nude mouse (6 weeks old, 20–23 g) in middle axillary lateral skin (n = 7 each group). We euthanized nude mice after 2 weeks, isolated xenograft tumors from colorectal cancer nude mice, and weighed them.

### 2.6 Cellular immunofluorescence

A total of 1 × 10^5^ cells were seeded into Lab-Tek cell culture well ((155,411, Thermo Scientific). After different treatments, we removed the cell culture medium and washed cells twice with PBS buffer. After being fixed and blocked, cells were incubated with GLUT1 antibody (ab115730, Abcam) or UBE2Q1 antibody (bs-24,324 R, Bioss) overnight at 4°C. The next day, we washed the cells 3 times with PBS buffer before incubating them with Alexa Fluor488 goat-anti-rabbit IgG (H + L) (A11008, Invitrogen). For cells, it should be counterstained the nucleus with 5 μg/mL DAPI for 5 minutes at room temperature. At last, all samples were analyzed by confocal microscopy (TCS SP5, Leica).

### 2.7 Lactate production and glucose uptake assay

After 24 hours of culture under normoxic (21% O2) or hypoxic conditions (1% O2), a cell culture medium was collected to detect the levels of lactate using a PicoProbe™ Lactate Fluorometric Assay Kit (K638, BioVision), and to detect the intracellular glucose levels using a PicoProbe™ Glucose Fluorometric Assay Kit (K688, BioVision).

### 2.9 Western blot analysis

Cells were harvested to extract total protein a Tissue/cells Protein Extraction Reagent (78,510, ThermoFisher). To determine the protein concentration, a BCA kit(23,227, ThermoFisher, USA) is used. Next, 50 μg total protein is analyzed using a 10% SDS-PAGE. After transferring, PVDF membranes (LC2002, ThermoFisher) are first blocked with 5% skimmed milk powder and then are probed with primary antibodies against UBE2Q1 (bs-24,324 R, Bioss), HIF-1α (ab279654, Abcam), or β-catenin (ab32572, Abcam). Proteins were visualized with ECL solution (WBKLS0100, Beijing Xinjingke Biotechnologies Co., Ltd, China), followed by densitometry analysis using Imag J 3.0 (IBM, USA) and β-actin was loading as control.

### 2.10 Statistical analysis

Data in this study were analyzed by SPSS 20.0 software. Data shown are mean ±SD. The difference between the two groups was compared by Student's t-test. For multiple groups, one-way ANOVA was used to compare the differences between multiple groups and then use the Turkey test as a post-doc test to compare the differences between the two groups. P-value less than 0.05 indicated a significant difference.

## 3 Results

In the present study, the main work is about glycolysis in colorectal cancer cells under hypoxia. Our goal is to discover a new mechanism for LINC00525 to regulate the Warburg effect of colorectal cancer cells under hypoxia. Fortunately, we not only found that LINC00525, which was highly expressed in colorectal cancer cells, promoted the proliferation of colorectal cancer cells in vivo and in vitro but also found that LINC00525 activated HIF-1α through the miR-338-3p/UBE2Q1/β-catenin axis to enhabcehypoxia-enhanced glycolysis in colorectal cancer.

### 3.1 LINC00525 is up-regulated and promotes cell proliferation in colorectal cancer

**Figure 1.**
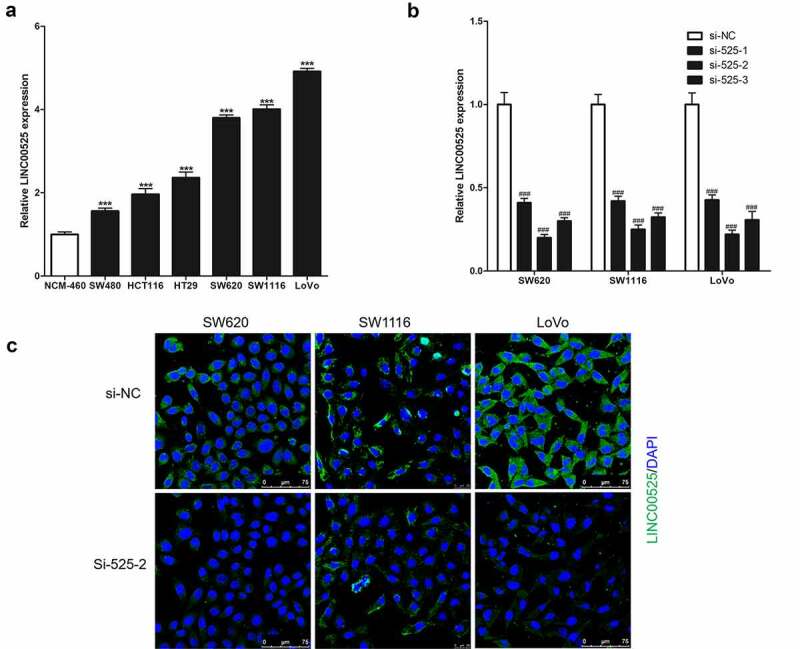
Expression of LINC00525 in colorectal cancer cells.

As a result of analysis in GSE110715, GSE32323, and GSE21510 datasets [12], LINC00525 was highly expressed in colorectal cancer tissues. In this study, it was found that the expression of LINC00525 in human colorectal cancer cells (SW480, HCT116, HT29, SW620, SW1116, and LoVo) were all markedly higher than its expression in human normal colonic epithelial cells (NCM-460) (Figure 1(a)). To investigate the function of LINC00525, the expression of LINC00525 was knocked down in human colorectal cancer cells (SW620, SW1116, and LoVo) by transferring into LINC00525-specific small interfering RNA (si-525-1, si-525-2, and si-525-3), and meaningless small interfering RNA (si-NC) was used as a negative control. As shown in Figure 1b, si-525-1, si-525-2, and si-525-3 all strongly reduced the expression of LINC00525 in colorectal cancer cells, with si-525-2 having the highest knockdown effect of the three siRNAs, decreasing LINC00525 expression to less than 25%. Similarly, the results of FISH staining also showed that si-525-1, si-525-2, and si-525-3 were all strongly knockdown the expression of LINC00525 in colorectal cancer cells, and si-525-2 has the best knockdown effect (Figure 1(c)). Therefore, if there were no special instructions, LINC00525-specific small interfering RNA (si-525) was si-525-2 in the following research. In addition, FISH staining analysis also showed that LINC00525 was mainly cytoplasmic localization in colorectal cancer cells (Figure 1(c)).

(A) Results of qPCR indicated that LINC0052 was highly expressed in colorectal cancer cells (SW480, HCT116, HT29, SW620, SW1116 and LoVo) which was compared with normal colonic epithelial cells (NCM-460). Data shown are mean ±SD (n = 3). *** P < 0.001 vs NCM-460 group.(B) LINC00525-specific small interfering RNA (si-525-1, si-525-2 and si-525-3) was transferred into colorectal cancer cells to knock down the expression of LINC00525, and meaningless small interfering RNA (si-NC) was used as a negative control. And qPCR analysis of the indicated LINC00525 levels significantly decreased after being transferred into si-525. Data shown are mean ±SD (n = 3). ### P < 0.001 vs si-NC group.(C) Results of FISH staining not only showed that LINC00525 was expressed in the cytoplasm of colorectal cancer cells, but also confirmed that si-525 significantly knocks down the expression of LINC00525.

**Figure 2.**
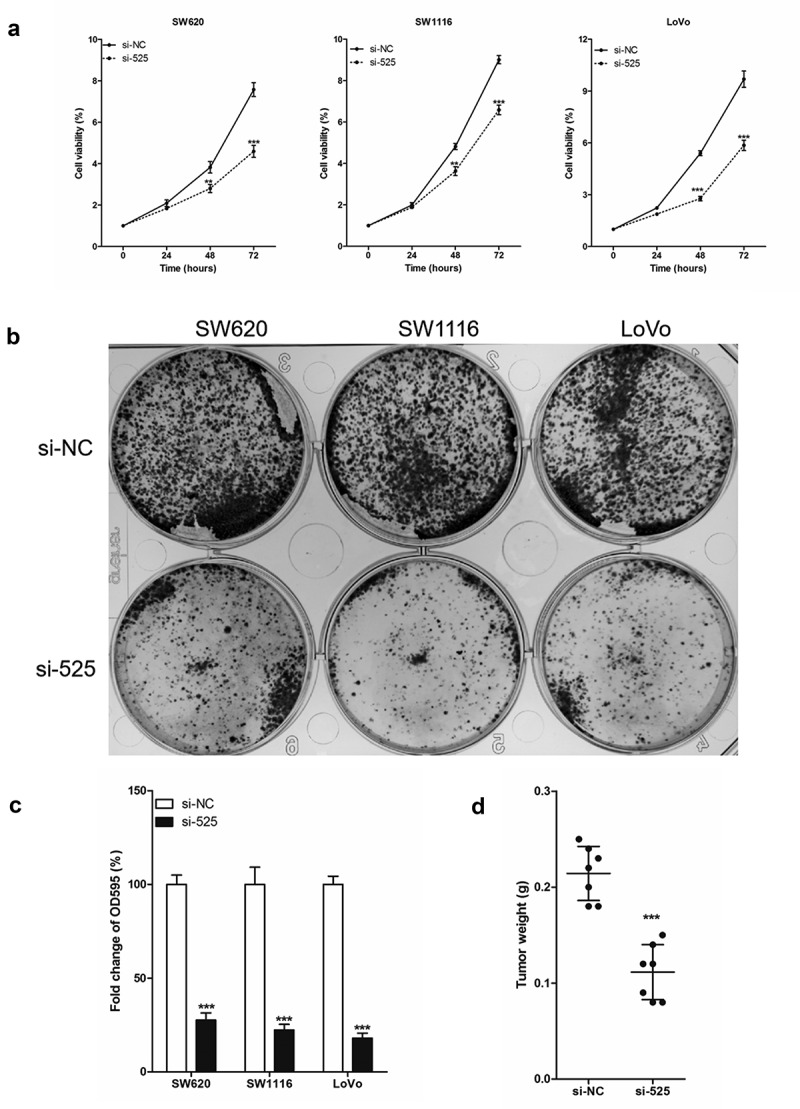
LINC00525 promoted the proliferation of colorectal cancer cells in vitro and in vivo.

Simultaneously, the effect of LINC00525 expression on the proliferation of colorectal cancer cells was explored with revealed that as compared to the colorectal cancer cells of the si-NC group, the cell viability of the si-525 group was significantly reduced in the 3-day culture experiment (Figure 2(a)). Similarly, the results of the cell clone formation experiment also showed that the number of colorectal cancer cell clones in the si-525 group was significantly were significantly less than that in the si-NC group (Figure 2(b-c)). In vivo, the weights of LoVo xenografts harvested 3 weeks after injection in the si-525 group were significantly lower than that in the si-NC group (Figure 2d). In conclusion, LINC00525 was highly expressed in colorectal cancer cells, and LINC00525 knockdown reduced the proliferation of colorectal cancer cells.

(A) Cell viability was detected in SW620, SW1116 and LoVo colorectal cancer cell knocking down LINC00525. Data shown are mean ±SD (n = 3). ** P < 0.01 and *** P < 0.001 vs si-NC group.(B) Representative photographs of cell clone were taken 2 weeks after culture.(C) Comparison of the number of cell clones in SW620, SW1116 and LoVo colorectal cancer cell knocking down LINC00525. Data shown are mean ±SD (n = 3).*** P < 0.001 vs si-NC group.(D) Comparison of the weights of xenografts harvested 3 weeks after injection (n = 7). Data shown are mean ±SD (n = 3). *** P < 0.001 vs si-NC group.

### 3.2 LINC00525 is essential for hypoxia-enhanced glycolysis

**Figure 3.**
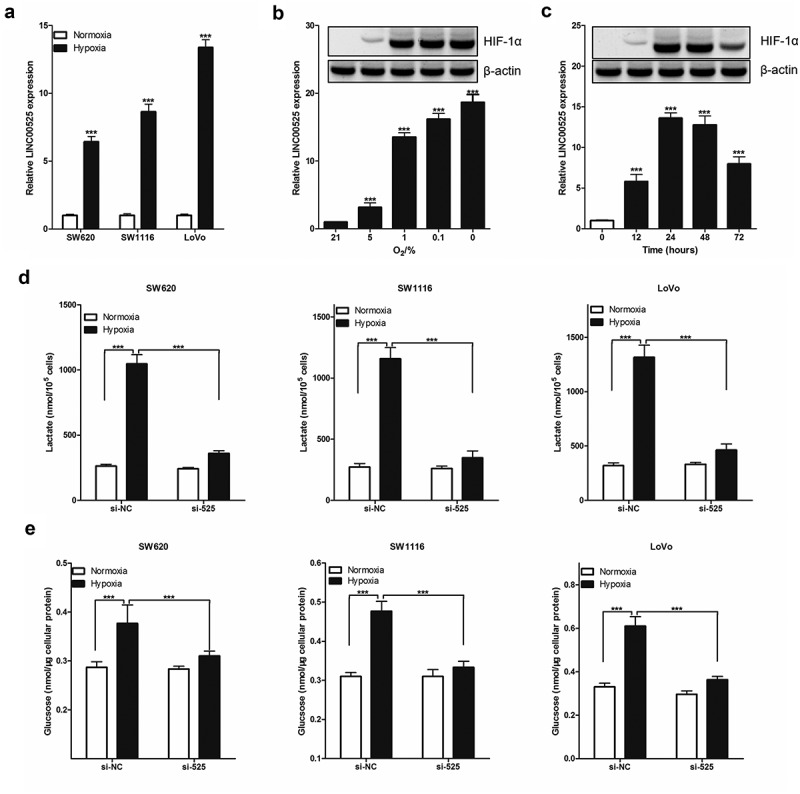
LINC00525 promoted hypoxic glycolysis in colorectal cancer cells.

One of the features of tumor cells is their dependence on glycolytic energy supply. To study whether LINC00525 regulates the Warburg effect under hypoxic conditions, we first assessed the amount of LINC00525 in colorectal cancer cells (SW620, SW1116, and LoVo) using RT-qPCR analysis. As shown in Figure 3(a), the expression of LINC00525 in colorectal cancer cells under hypoxic conditions (1% O_2_ for 24 hours) was significantly higher than that in colorectal cancer cells under normoxic conditions (21% O_2_ for 24 hours). Moreover, we also study the effect of different hypoxia conditions on the expression of LINC00525 in colorectal cancer cells (LoVo) and found that LINC00525 expression increased as the oxygen concentration decreased (Figure 3(b)), and also increased as with the duration of hypoxia (Figure 3(c)). At the same time, the expression of HIF-1α protein was also detected which showed that its expression was also related to hypoxia in a concentration-dependent and time-dependent manner.

(A) 24 hours after being cultured under normoxic (21% O_2_) or hypoxic conditions (1% O_2_), SW620, SW1116 and LoVo were harvested to detect LINC00525 expression using qPCR. Data shown are mean ±SD (n = 3). *** P < 0.001 vs normoxia group.(B) LoVo cells were cultured under the indicated conditions for 24 hours, and then being harvested to detect LINC00525 expression using qPCR and detect HIF-1α using immunoblotting. Data shown are mean ±SD (n = 3). *** P < 0.001 vs normoxia group (21% O_2_ group).(C) LoVo cells were cultured under hypoxic conditions (1% O_2_) for different time, and then being harvested to detect LINC00525 expression using qPCR and detect HIF-1α using immunoblotting. Data shown are mean ±SD (n = 3). *** P < 0.001 vs normoxia group (0 hour group).(D) 24 hours after being cultured under normoxia or hypoxia, we compared lactate levels in the culture medium of SW620, SW1116 and LoVo cells. Data shown are mean ±SD (n = 3). *** P < 0.001 between two groups.(E) 24 hours after being cultured under normoxia or hypoxia, we compared intracellular glucose levels of SW620, SW1116 and LoVo cells. Data shown are mean ±SD (n = 3). *** P < 0.001 between two groups.

To investigate the functional role of hypoxia-induced LINC00525, we knocked down the expression of LINC00525 in colorectal cancer cells (SW620, SW1116, and LoVo) by transferring into si-525. It has been well recognized that hypoxia causes increased glycolysis. Treatment with hypoxia resulted in a significant increase in lactate production (Figure 3(d)) and glucose uptake (Figure 3(e)). All these hypoxia-induced effects, however, were greatly reversed by LINC00525 knockdown (Figure 3(d-e)), indicating that LINC00525 functions as a critical mediator of hypoxia-enhanced glycolysis.

### 3.3 HIF-1α is necessary for the effect of LINC00525 on hypoxia-enhanced glycolysis

**Figure 4.**
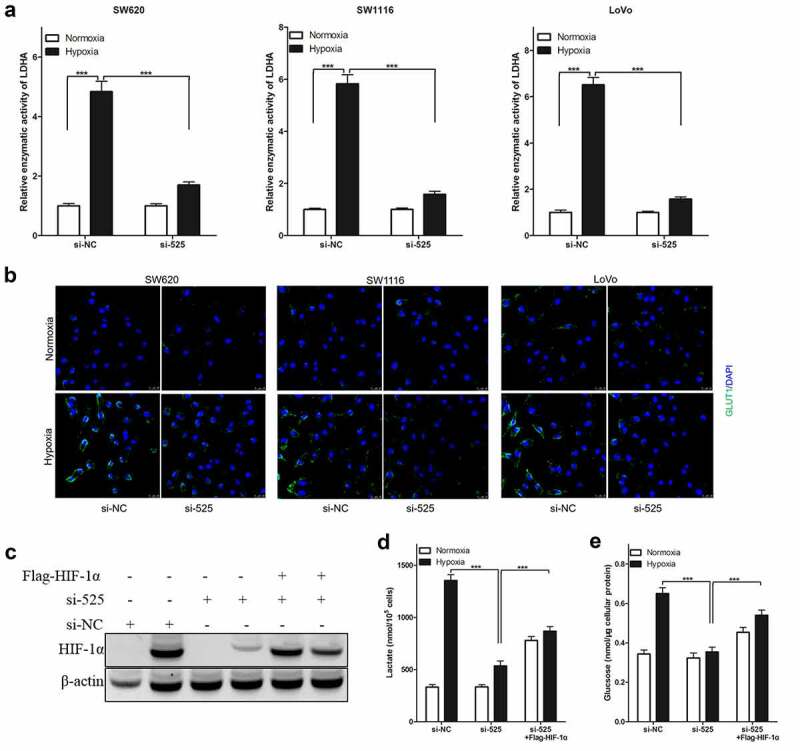
Effect of LINC00525 on hypoxic glycolysis is HIF-1α dependent.

In colorectal cancer cells, LINC00525 knockdown significantly reduced hypoxia-enhanced lactate production (Figure 3(d)) and glucose uptake (Figure 3(e)) (SW620, SW1116, and LoVo), similar to the above findings. It was also found that LINC00525 knockdown significantly decreased the relative enzymatic activity of LDHA (Figure 4(a)) and GLUT1 protein expression (Figure 4(b)) in colorectal cancer cells (SW620, SW1116, and LoVo) after being cultured under hypoxia. Due to HIF-1α being necessary for hypoxia-enhanced LDHA activity and GLUT1 expression, whether LINC00525 promoted hypoxic-enhanced glycolysis by regulating HIF-1α was investigated. Firstly, it was found that LINC00525 knockdown decreased the expression of HIF-1α in LoVo cells after being cultured under hypoxia (Figure 4(c)). Therefore, to study whether HIF-1α is necessary for the effect of LINC00525 on hypoxia-enhanced glycolysis, exogenous HIF-1α was introduced into LINC00525 knockdown LoVo cells, which upregulated the expression of HIF-1α protein. The results of immunoblotting showed that Flag-HIF-1α increased the expression of HIF-1α protein in LINC00525 knockdown LoVo cells (Figure 4(c)). Next, the glycolysis in LoVo cells was assessed. Overexpression of HIF-1a indeed reversed the effect of LINC00525 on lactate production (Figure 4(d)) and glucose uptake (Figure 4(e)). Therefore, our data indicated that the effect of LINC00525 on hypoxic glycolysis was HIF-1α dependent.

(A) 24 hours after being cultured under normoxia or hypoxia, the enzymatic activity of LDHA in SW620, SW1116 and LoVo cells were measured and compared. Data shown are mean ±SD (n = 3). *** P < 0.001 between two groups.(B) 24 hours after being cultured under normoxia or hypoxia, and then analyzed GLUT1 expression using immunofluorescence.(C) LoVo cells expressing either si-NC or si-525 were transfected with or without Flag-HIF-1α, as indicated. 24 hours later, LoVo cells were cultured under normoxia or hypoxia for another 24 hours, and then being harvested to detect HIF-1α expression using immunoblotting.(D) LoVo cells expressing either si-NC or si-525 were transfected with or without Flag-HIF-1α, as indicated. 24 hours later, LoVo cells were cultured under normoxia or hypoxia for another 24 hours, we compared lactate levels in the culture medium of LoVo. Data shown are mean ±SD (n = 3). *** P < 0.001 between two groups.(E) LoVo cells expressing either si-NC or si-525 were transfected with or without Flag-HIF-1α, as indicated. 24 hours later, LoVo cells were cultured under normoxia or hypoxia for another 24 hours, we compared intracellular glucose levels of LoVo. Data shown are mean ±SD (n = 3). *** P < 0.001 between two groups.

### 3.4 LINC00525 promotes UBE2Q1 expression by binding miR-338-3p

**Figure 5.**
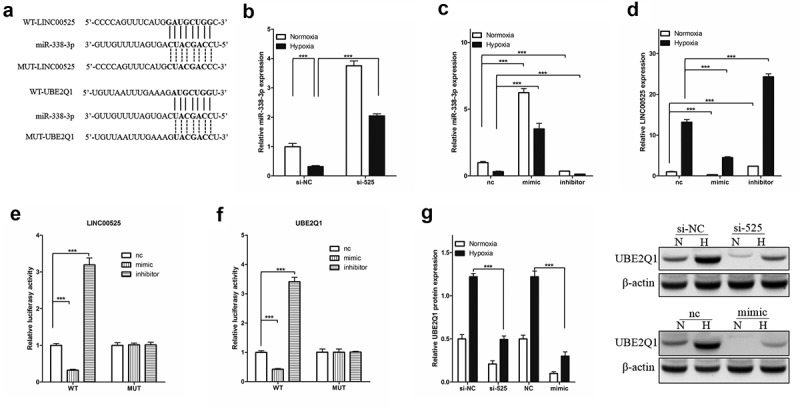
LINC00525 promoted UBE2Q1 expression by sponging to miR-338-3p.

The public databases (UCSC, miRbase, and BiBiserv2) were used to gain insight into the specific molecular mechanism of LINC00525 in hypoxic glycolysis. Interestingly, it was found that miR-338-3p, a Warburg effect-related miRNA [18] and a tumor suppressor gene in colorectal cancer cells [14,28]. Interestingly, we found that miR-338-3p, LINC00525, and UBE2Q1 all have mutually binding sequences (Figure 5(a)). As shown in Figure 5(b), LINC00525 knockdown significantly increased the expression of miR-338-3p in LoVo cells under normoxic and hypoxic conditions (Figure 5(b). Next, miR-338-3p expression was enhanced by transferring into miR-338-3p-mimic (mimic) and knockdown miR-338-3p expression by transferring into miR-338-3p-inhibitor (inhibitor) in LoVo cells and confirmed by qPCR (Figure 5(c)). Importantly, it was found that under normoxic and hypoxic conditions, mimic significantly decreased LINC00525 expression whereas inhibitor significantly enhanced LINC00525 expression in LoVo cells (Figure 5(d)), which suggests LINC00525 and miR-338-3p reduce the expression of each other by binding. Consistent with the above findings that miR-338-3p inhibited LINC00525 expression in LoVo cells, the dual fluorescent gene reporting system showed the luciferase activity of LINC00525-wt was significantly decreased by mimic, while miR-338-3p had no influence on LINC00525-Mut in LoVo cells, suggesting miR-338-3p directly inhibited LINC00525 expression (Figure 5(e)). At the same time, the results of the dual fluorescent gene reporting system also showed that miR-338-3p (Figure 5(e)). Following immunoblotting results, LINC00525 knockdown significantly decreased UBE2Q1 protein expression, while miR-338-3p overexpression also significantly decreased UBE2Q1 protein expression in LoVo cells under normoxic and hypoxic conditions (figure 5(f)).

(A) Schematic diagram of binding sites of the 3’UTR of LINC00525 (up) or UBE2Q1 mRNA (down) and miR-338-3p.(B) LoVo cells expressing either si-NC or si-525 were transfected, as indicated. 24 hours later, LoVo cells were cultured under normoxia or hypoxia for another 24 hours, and then being harvested to detect miR-338-3p expression. Data shown are mean ±SD (n = 3). *** P < 0.001 between two groups.(C and D) LoVo cells expressing either miR-338-3p-nc (nc), miR-338-3p-mimic (mimic) or miR-338-3p-inhibitor (inhibitor), as indicated. 24 hours later, LoVo cells were cultured under normoxia or hypoxia for another 24 hours, and then being harvested to detect miR-338-3p (C) or LINC00525 (D) expression. Data shown are mean ±SD (n = 3). *** P < 0.001 between two groups.(E) LoVo cells expressing either miR-338-3p-nc (nc), miR-338-3p-mimic (mimic) or miR-338-3p-inhibitor (inhibitor) were transfected with LINC00525 wild type (WT) or mutant (MUT) transcript, and then detected luciferase activity. Data shown are mean ±SD (n = 3). *** P < 0.001 between two groups.(F) LoVo cells expressing either miR-338-3p-nc (nc), miR-338-3p-mimic (mimic) or miR-338-3p-inhibitor (inhibitor) were transfected with UBE2Q1 wild type (WT) or mutant (MUT) transcript, and then detected luciferase activity. Data shown are mean ±SD (n = 3). *** P < 0.001 between two groups.(G) LoVo cells expressing either si-NC, si-525, miR-338-3p-nc (nc) or miR-338-3p-mimic (mimic), as indicated. 24 hours later, LoVo cells were cultured under normoxia or hypoxia for another 24 hours, and then being harvested to detect UBE2Q1 protein expression. Data shown are mean ±SD (n = 3). *** P < 0.001 between two groups.

### 3.5 LINC00525 promotes hypoxia-enhanced glycolysis is UBE2Q1 dependent

**Figure 6.**
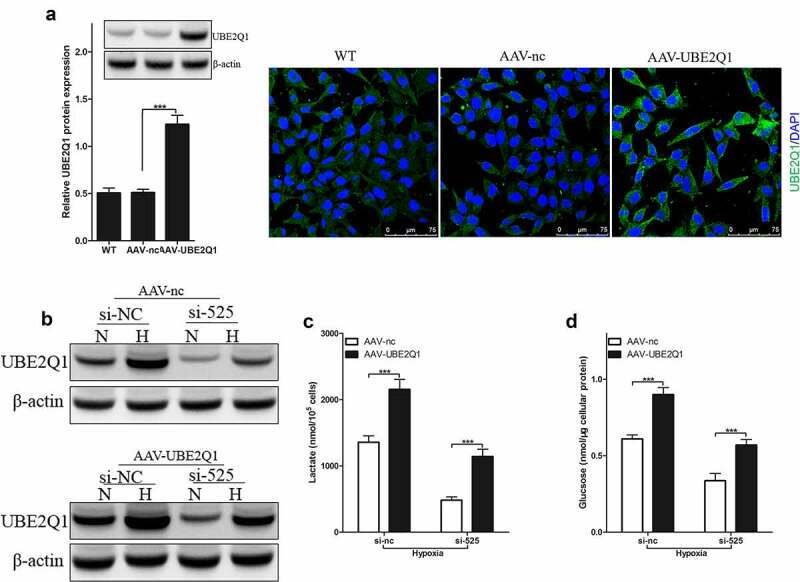
Effect of LINC00525 on hypoxic glycolysis is UBE2Q1 dependent.

UBE2Q1, whose full name is ubiquitin-conjugating enzyme E2Q family member 1, was found to be highly expressed in colorectal cancer tumor tissues and cells [29]. To investigate the effect of UBE2Q1 on hypoxia-enhanced glycolysis, we infected LoVo cells with AAV-UBE2Q1 adenovirus and found that immunoblotting and immunofluorescence significantly increased the expression of UBE2Q1 in LoVo cells (Figure 6(a)). LINC00525 knockdown LoVo cells were infected with AAV-UBE2Q1 to upregulated UBE2Q1 protein expression.

(A) LoVo cells infecting either AAV-nc or AAV-UBE2Q1 adenovirus, as indicated. 48 hours later, we harvested LoVo cells to detect UBE2Q1 protein expression using immunoblotting (left) and immunofluorescence (right). Data shown are mean ±SD (n = 3). *** P < 0.001 between two groups.(B) LoVo cells expressing either si-NC or si-525 were infected with AAV-nc or AAV-UBE2Q1 adenovirus as indicated. 24 hours later, LoVo cells were cultured under normoxia or hypoxia for another 24 hours, and then being harvested to detect UBE2Q1 protein expression using immunoblotting.(C) LoVo cells expressing either si-NC or si-525 were infected with AAV-nc or AAV-UBE2Q1 adenovirus as indicated. 24 hours later, LoVo cells were cultured under hypoxia for another 24 hours, we compared lactate levels in the culture medium of LoVo. Data shown are mean ±SD (n = 3). *** P < 0.001 between two groups.(D) LoVo cells expressing either si-NC or si-525 were infected with AAV-nc or AAV-UBE2Q1 adenovirus as indicated. 24 hours later, LoVo cells were cultured under hypoxia for another 24 hours, we compared intracellular glucose levels of LoVo. Data shown are mean ±SD (n = 3). *** P < 0.001 between two groups.

Results of immunoblotting showed that AAV-UBE2Q1 increased the expression of UBE2Q1 protein in LINC00525 knockdown LoVo cells (Figure 6(b)). Then, glycolysis in LoVo cells was checked. Overexpression of UBE2Q1 indeed reversed the effect of LINC00525 on lactate production (Figure 6(c)) and glucose uptake (Figure 6(c)). Therefore, these data indicated that the effect of LINC00525 on hypoxic glycolysis was UBE2Q1 dependent.

## 3.6 UBE2Q1enhanced hypoxia-enhanced glycolysis by stabilizing β-catenin

**Figure 7.**
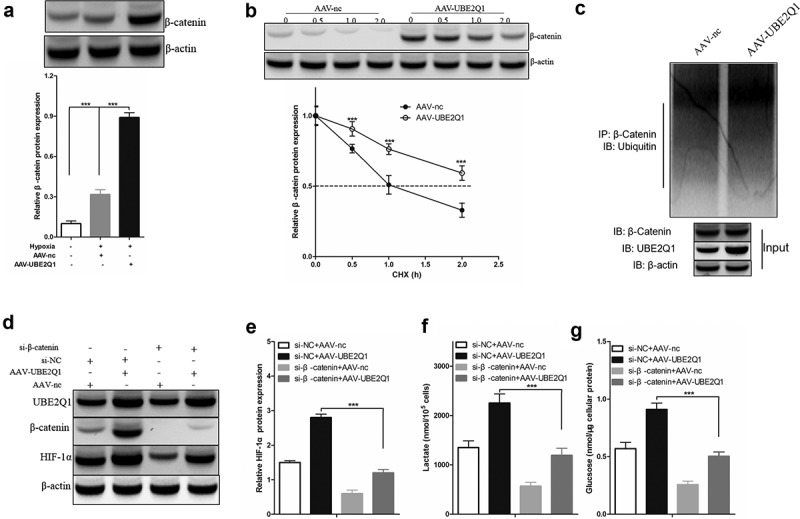
UBE2Q1 stabilized β-catenin, and β-catenin knockdown hypoxia-induced abrogated LINC00525-augmented HIF-1α activation and glycolysis.

Overexpression of UBE2Q1 reversed the effect of LINC00525 on hypoxia-enhanced glycolysis, as seen above. Overexpression of UBE2Q1 specifically enhanced hypoxia-enhanced glycolysis. However, so far, its molecular mechanism is still unknown. In a previous study, UBE2Q1 has found to stabilize β-catenin in hepatocellular carcinoma [21], and β-catenin interacts with HIF-1α under hypoxic conditions [22]. These clues hint that UBE2Q1-stabilized β-catenin enhances hypoxia-enhanced glycolysis by activating HIF-1α. To test this hypothesis, we first found that overexpression of UBE2Q1 significantly β-catenin protein expression in LoVo cells under hypoxia (Figure 7(a)). In addition, cycloheximide (CHX) tracking experiments showed that the half-life of β-Catenin was prolonged in LoVo cells overexpressing UBE2Q1 (Figure 7BAt the same time, we observed that overexpression of UBE2Q1 significantly enhanced-catenin ubiquitination in LoVo cells (Figure 7(c)). To study the effect of β-catenin on the effect of UBE2Q1 on hypoxia-enhanced glycolysis, we used si-β-catenin to knock down the expression of β-catenin in UBE2Q1 overexpression LoVo cells (Figure 7(d)). Importantly, it was found that β-catenin knockdown reversed the effect of UBE2Q1 on HIF-1α activation (Figure 7(e)), lactate production (Figure 7(f)), and glucose uptake (Figure 7(g)). Therefore, these results indicated that UBE2Q1-stabilized β-catenin enhances hypoxia-enhanced glycolysis by activating HIF-1α.

(A) LoVo cells infecting either AAV-nc or AAV-UBE2Q1 adenovirus, as indicated. 24 hours later, LoVo cells were cultured under normoxia or hypoxia for another 24 hours, and then being harvested to detect UBE2Q1 protein expression using immunoblotting. Data shown are mean ±SD (n = 3). *** P < 0.001 between two groups.(B) LoVo cells infecting either AAV-nc or AAV-UBE2Q1 adenovirus were treated with cycloheximide (CHX) for increasing time periods, as indicated. and then being harvested to detect β-catenin protein expression using immunoblotting. Data shown are mean ±SD (n = 3). *** P < 0.001 between two groups. Data shown are mean ±SD (n = 3). *** P < 0.001 between two groups.(C) The lysates of LoVo stably overexpressing AAV-nc or AAV-UBE2Q1 adenovirus were immunoprecipitated (IP) with β-Catenin antibody. The IP products were further analyzed by immunoblotting to detect ubiquitin expression. The expression of β-Catenin and UBE2Q1 in the input fraction is presented below.(D) LoVo cells expressing either si-NC or si-525 were infected with AAV-nc or AAV-UBE2Q1 adenovirus as indicated. 24 hours later, LoVo cells were cultured under hypoxia for another 24 hours, and then being harvested to detect UBE2Q1, β-Catenin and HIF-1α protein expression using immunoblotting.(E) Comparison of HIF-1α protein expression in LoVo cells which was treated as showed in (D). Data shown are mean ±SD (n = 3). *** P < 0.001 between two groups.(F) LoVo cells expressing either si-NC or si-525 were infected with AAV-nc or AAV-UBE2Q1 adenovirus as indicated. 24 hours later, LoVo cells were cultured under hypoxia for another 24 hours, we compared lactate levels in the culture medium of LoVo. Data shown are mean ±SD (n = 3). *** P < 0.001 between two groups.(G) LoVo cells expressing either si-NC or si-525 were infected with AAV-nc or AAV-UBE2Q1 adenovirus as indicated. 24 hours later, LoVo cells were cultured under hypoxia for another 24 hours, we compared intracellular glucose levels of LoVo. Data shown are mean ±SD (n = 3). *** P < 0.001 between two groups.

## 4. Discussion

In this study, it was found that LINC00525 was highly expressed and promoted cell proliferation in colorectal cancer, which has been found in previous studies [11,13]. This study focused on the Warburg effect as it is a key factor in tumor pathogenesis 1 and provides a microenvironment suitable for tumor cells to survive, proliferate, and move [2,3]. More importantly is that there is no research between LINC00525 and the Warburg effect of colorectal cancer cells, which guarantees the innovation of research.

In the present study, it was found for the first time that LINC00525 knockdown decreased hypoxia-enhanced glycolysis, and overexpression of HIF-1α reversed the effect of LINC00525 knockdown on hypoxia-enhanced glycolysis in colorectal cancer, which suggests that LINC00525 promoted hypoxia-enhanced glycolysis, and this effect was HIF-1α dependent. Previous studies have shown that under hypoxic conditions, the activation of HIF-1α contributes to the Warburg effect by synergistically up-regulating glycolysis and down-regulating oxidative phosphorylation. It followed this mechanism: increase the uptake of glucose by cancer cells by up-regulating the expression of GLUT1 [30,31], and speed up the metabolism from glucose to pyruvate, and promote the conversion of pyruvate to lactic acid by up-regulating the expression of LDHA [32,33].

Furthermore, we found that LINC00525 is mostly present in the cytoplasm of colorectal cancer cells. The localization of lncRNA in cells is well known to be closely related to its mechanism of action. The ceRNA regulation mechanism is one of the important ways for lncRNA to regulate post-transcription in the cytoplasm. Previous studies have found that lncRNA can be used as the ‘sponge’ of miRNA, competitively binding to miRNA, thereby affecting the regulation of miRNA on downstream target genes [6,32]. Therefore, the core of the cytoplasm transcriptional regulation mechanism of lncRNA is that lncRNA and a specific mRNA share a miRNA. miR-338-3p was identified as a downstream gene of LINC00525 through literature search and public databases to study the molecular mechanism of LINC00525 regulating hypoxic glycolysis in colorectal cancer cells. miR-338-3p was identified as a downstream gene of LINC00525 to study hypoxic glycolysis because miR-338-3p was found to inhibit the proliferation and migration of colorectal cancer cells [14,15], as well as it was observed to be involved in the regulation of tumor cell glycolysis ^1819^. Herein, it was found that LINC00525 and miR-338-3p reduced the expression of each other by binding, and miR-338-3p overexpression and LINC00525 knockdown inhibited UBE2Q1 protein expression in colorectal cancer cells.

UBE2Q1 is found on the long arm of chromosome 1 and encodes the ubiquitinated ligase E2 protein. UBE2Q1 moves activated ubiquitin from the ubiquitin-activating enzyme (E1) to the ubiquitin ligase (E3) and the substrate, activating the substrate’s role to degrade ubiquitin ^33^. The previous study has found that UBE2Q1 was highly expressed in colorectal cancer [29], and increased the levels of β-catenin protein by stabilizing β-catenin in cancer cells [21], and β-catenin interacts with HIF-1α under hypoxic condition [22]. Consistently, it was found that UBE2Q1 overexpression increased the expression of β-catenin and also increased the ubiquitination level of β-catenin and elongated its half-life in colorectal cancer cells. Moreover, it was found that β-Catenin knockdown removed the enhanced expression of HIF-1α by UBE2Q1 overexpression and reversed the effect of UBE2Q1 on lactate production and glucose uptake in colorectal cancer under hypoxia, suggesting that UBE2Q1-stabilized β-catenin enhances hypoxia-enhanced glycolysis by activating HIF-1α.

## Conclusion

It can be concluded that LINC00525 promoted UBE2Q1 expression by binding miR-338-3p, and UBE2Q1-stabilized β-catenin enhances hypoxia-enhanced glycolysis by activating HIF-1α. Understanding how LINC00525 participates in the regulation of the Warburg effect in colorectal cancer provides novel insights on the issue.

## Data Availability

The data used to support the findings of this study are available from the corresponding author upon request.
